# Circulating Endoglin Concentration Is Not Elevated in Chronic Kidney Disease

**DOI:** 10.1371/journal.pone.0023718

**Published:** 2011-08-19

**Authors:** David M. Charytan, Alexander M. Helfand, Brian A. MacDonald, Angeles Cinelli, Raghu Kalluri, Elisabeth M. Zeisberg

**Affiliations:** 1 Renal Division, Department of Medicine, Brigham and Women's Hospital, Boston, Massachusetts, United States of America; 2 Division of Matrix Biology, Department of Medicine, Beth Israel Deaconess Medical Center, Boston, Massachusetts, United States of America; Biomedical Research Foundation of the Academy of Athens, Greece

## Abstract

**Background:**

Soluble endoglin, a TGF-β receptor, plays a key role in cardiovascular physiology. Whether circulating concentrations of soluble endoglin are elevated in CKD or underlie the high risk of cardiovascular death associated with chronic kidney disease (CKD) is unknown.

**Methods:**

Individuals with and without CKD were recruited at a single center. Estimated glomerular filtration rate (eGFR) was estimated using the modified MDRD study equation and the serum creatinine at the time of recruitment, and patients were assigned to specific CKD stage according to usual guidelines. Serum endoglin concentration was measured by ELISA and univariate and multivariable regression was used to analyze the association between eGFR or CKD stage and the concentration of soluble endoglin.

**Results:**

Serum endoglin was measured in 216 patients including 118 with stage 3 or higher CKD and 9 individuals with end stage renal disease (ESRD). Serum endoglin concentration did not vary significantly with CKD stage (increase of 0.16 ng/mL per 1 stage increase in CKD, P = 0.09) or eGFR (decrease -0.06 ng/mL per 10 mL/min/1.73 m^2^ increase in GFR, P = 0.12), and was not higher in individuals with ESRD than in individuals with preserved renal function (4.2±1.1 and 4.3±1.2 ng/mL, respectively). Endoglin concentration was also not significantly associated with urinary albumin excretion.

**Conclusions:**

Renal function is not associated with the circulating concentration of soluble endoglin. Elevations in soluble endoglin concentration are unlikely to contribute to the progression of CKD or the predisposition of individuals with CKD to develop cardiovascular disease.

## Introduction

Angiogenesis plays a critical role in renal homeostasis, and emerging data have increasingly suggested that dysregulation of angiogenesis contributes to the progression of chronic kidney disease (CKD) and its associated complications[1], [2], [3], [4], [5], [6], [7], [8], [9]. Increases in the circulating levels of angiogenesis inhibitors may play an important role in the rarefaction of renal capillaries that accompanies uremia[8]. Similarly, a wealth of data has demonstrated that vascular endothelial growth factor (VEGF) signaling plays a critical role in the maintenance of normal glomerular function[1], [5], [7], [10], and that inhibiting VEGF induces hypertension, proteinuria and significant pathologic changes within the glomerulus [6], [7], [10], [11].

The auto-regulation of angiogenesis plays a similarly important role in cardiovascular homeostasis [12], [13], [14]. Inhibition of VEGF signaling, for example, induces interstitial inflammation and endothelial apoptosis in the heart [15], [16], and in both animal models and human CKD uremia is accompanied by significant rarefaction of the myocardial microvasculature[12], [13], [17]. These observations suggest that circulating anti-angiogenic factors may contribute to the progression of CKD and that their concentrations are likely to be elevated in individuals with significant loss of glomerular filtration rate (GFR) or proteinuria. Furthermore, Elevations in the ambient level of circulating angiogenesis inhibitors in individuals with CKD would also provide a mechanistic explanation for the high risk of cardiovascular morbidity and mortality associated with CKD.

Although, two recent studies have demonstrated that soluble fms-like kinase (sFLT-1) concentration rises as GFR declines [18], [19], it remains uncertain whether angiogenesis inhibitors are generally elevated in the setting of CKD or whether sFLT-1 is uniquely increased in this respect. In particular, it is unknown whether the concentration of soluble endoglin, a key angiogenesis inhibitor implicated in the pathogenesis of preeclampsia cardiovascular disease, hypertension and endothelial dysfunction [20], [21], [22], [23], is correlated with GFR. Because endoglin appears to play key roles in the pathogenesis of renal and cardiovascular pathology, we hypothesized that elevations in the concentration of circulating endoglin in CKD could contribute to the progression of CKD and underlie the association of CKD and cardiovascular disease. We undertook this study in order to determine whether endoglin levels are higher in individuals with CKD compared to individuals with preserved renal function.

## Methods

### Ethics Statement

This research was approved by and conducted in accordance with the standards of the local institutional review committees of the Brigham & Women's Hospital and Beth Israel Deaconess Medical Center and the principles of the Declaration of Helsinki. All subjects involved in the research signed written informed consent forms prior to enrollment.

### Subjects

Subjects with and without CKD between the ages of 18–80 years old were recruited from the cardiac catheterization lab and the outpatient nephrology clinics (including the general nephrology, stone, and renal transplant donor clinics) of Brigham and Women's Hospital. Individuals receiving anti-angiogenic or immunosuppressive therapy, those with active malignancy or acute coronary ischemia, a history of thoracic or abdominal radiation therapy, and individuals with acute kidney injury were excluded. Individuals undergoing angiography were additionally excluded if they had a history of prior coronary artery bypass surgery.

### Clinical Data and Laboratories

Data on blood pressure, demographics and baseline clinical conditions were obtained by patient interview, supplemental chart review and a brief exam at the time of recruitment. Serum creatinine, albumin, and hemoglobin were measured on the day of enrollment in the hospital laboratory using standard clinical techniques. The most recent measurements of total cholesterol, urinary albumin and creatinine excretion within 6 months of recruitment were also collected. Estimated glomerular filtration rate (eGFR) was calculated using the modified MDRD equation [24]. Renal function was categorized using a modification of the National Kidney Foundation 5-stage system [25]. Because the MDRD equation is not precise in patients with preserved renal function, individuals with eGFR ≥90 mL/min/1.73 m^2^ were combined into a single category (“preserved function/stage 1 CKD)—this category included all patient with eGFR≥90 mL/min/1.73 m^2^. As a sensitivity analysis, we separately analyzed endoglin concentrations in those whose GFR was estimated to be within the normal range (eGFR ≥110 mL/min/1.73 m^2^) [26] and in individuals who were seen as transplant donors or for renal stones (without concomitant CKD). Non-dialysis dependent stage 4 and 5 CKD were considered jointly, chronic dialysis patients were evaluated as a unique category.

### Samples and Measurement of Endoglin

Serum was centrifuged within 15 minutes at 1000 g and stored at −80°C. Samples were batch analyzed for soluble endoglin concentration in a blinded fashion using ELISA kits from R&D systems. Intra and inter-assay coefficient of variations for the assay were 4.0 and 13.1%, respectively.

### Statistical Analysis

Data are presented as mean±standard deviation (SD), n (%) or median (inter-quartile ratio, IQR). Differences in baseline characteristics such as demographic data, blood pressure and comorbid medical conditions across categories of renal function were compared using ANOVA or non-parametric trend tests for continuous data and Chi-Square tests of trend for count data. The Pearson correlation coefficient was used to measure correlation between continuous variables. The relationship between eGFR or categories of renal function and soluble endoglin was assessed using univariate linear regression and a series of 4 different multivariable regression models to correct for potential confounding. The primary comparison was performed using linear regression models with serum endoglin concentration as the dependent variable, renal function (either as a continuous or categorical variable) as the primary independent variable and with additional adjustment for age, sex, race, diabetes, hypertension, smoking, cholesterol, congestive heart failure, peripheral vascular disease, and obstructive lung disease and recruitment site (Model 1). An expanded linear regression model (Model 2) additionally adjusted for Hispanic ethnicity and serum albumin. Additional linear models including terms for interaction with diabetes and recruitment site (outpatient clinics vs. coronary catheterization lab) were performed as a pre-specified analysis. Two sensitivity analyses were performed in order to examine the robustness of our results and their sensitivity to modeling assumptions. In the first, the independent terms from Model 1 were used in an ordered categorical regression model using quartiles of endoglin as the outcome variable. Logistic regression models using the median value of endoglin as the cutoff were generated in an analogous fashion. Model fit was tested using graphical techniques, inspection of residual distribution, and tests of model specification. All analyses were performed in STATA 9.0 (Stata Corporation, College Station, TX). P<0.05 was considered significant for all analyses.

## Results

### Baseline Characteristics

As shown in Table 1 and Table 2, serum creatinine and eGFR were significantly different across categories of renal function: eGFR in preserved function/Stage 1 CKD 105.9±12.0 mg/dL, Stage 2 CKD 73.8±8.5, Stage 3 CKD 44.8±8.5, Stage 4/5 CKD 22.5±6.5, Dialysis , 8.9±2.1 ,P <0.001. Age increased across increasing categories of CKD, but was actually lower in individuals on dialysis (52.3±18.0 years) than in individuals with preserved renal function/stage 1 CKD (57.3±8.0). Among baseline comorbidities, only coronary artery disease (P = 0.003), peripheral vascular disease (P = 0.007), and diabetes (P = 0.01) demonstrated a significantly increasing frequency across categories of renal function. Congestive heart failure was more prevalent in individuals with more severe renal dysfunction, but this trend did not achieve significance (P = 0.07). Diastolic blood pressure increased from 74.0±12.9 in individuals with preserved function/Stage 1 CKD to 82.6±18.5 in individuals on dialysis (P = 0.02). Similar trends were observed with systolic blood pressure but were not significant.

**Table 1:** Baseline Clinical Characteristics of the Study Population.

Characteristic	Preserved Function-Stage 1 CKD	Stage 2 CKD	Stage 3 CKD	Stage 4-5 CKD	Dialysis	P Value*
(Mean±S.D.)	(N = 30)	(N = 5)	(N = 92)	(N = 27)	(N = 9)	
**Demographics**						
Age, years	57.3 ±8.0	59.8 ±11.3	63.5±13.2	68.2±14.7	52.3±18.0	0.001
Male, n (%)	22 (73.3)	38 (65.5)	49 (53.3)	18 (66.7)	3 (33.3)	0.10
Race, n (%)						
Black	3 (10.0)	9 (15.8)	10 (11.2)	4 (15.4)	4 (44.4)	0.06
White	25 (83.3)	42 (73.7)	68 (76.4)	18 (69.2)	2 (22.2)	
Other	2 (6.7)	6 (10.5)	11 (12.4)	4 (15.4)	3 (33.3)	
Hispanic	2 (6.7)	6 (10.3)	15 (16.5)	6 (24.0)	2 (22.2)	0.31
**Physical Exam**						
Weight, kilogram	86.1±19.2	86.8 ±21.7	89.8±23.8	85.5±20.0	81.9±32.7	0.87
Systolic blood pressure, mm Hg	128.0±24.2	124.7±17.3	128.9±21.4	130.2±19.7	146.4±13.8	0.08
Diastolic blood pressure, mm Hg	74.0±12.9	70.7±11.2	67.8±11.9	70.8±20.9	82.6±18.5	0.02
**Past Medical History,** n (%)						
Coronary disease	13 (76.5)	17 (43.6)	20 (26.0)	10 (38.5)	3 (42.9)	0.003
Hypertension	23 (76.7)	42 (72.4)	72 (78.3)	25 (92.6)	9 (100.0)	0.13
Diabetes	9 (30.0)	20 (34.5)	45 (48.9)	19 (70.4)	5 (55.6)	0.01
Congestive heart failure	4 (13.3)	11 (19.0)	16 (17.4)	6 (22.2)	5 (55.6)	0.07
Obstructive lung disease	0 (0.0)	2 (3.5)	6 (6.5)	1 (3.7)	2 (22.2)	0.10
Peripheral vascular disease	2 (6.7)	3 (5.2)	8 (8.7)	8 (29.6)	2 (22.2)	0.007
Hyperlipidemia	21 (70..0)	42 (72.4)	62 (67.4)	19 (70.4)	6 (66.7)	0.98
Smoking	3 (13.0)	9 (17.0)	7 (8.1)	0 (0.0)	1 (12.5)	0.17
Cause of CKD						0.29
Diabetes	--	--	13 (14.1)	8 (29.6)	1 (11.1)	
Hypertension	--	--	17 (18.5)	2 (7.4)	2 (22.2)	
Other/Unknown	--	--	62 (67.4)	17 (63.0)	6 (66.7)	

S.D. = Standard deviation., I.Q.R. = Interquartile Range,

*ANOVA for normally distributed continuous variables, chi-square test for -parametric trend test for non-normally distributed continuous variables, and chi-squared tests for count variables.

**Table 2:** Baseline Laboratory Characteristics of the Study Population.

Characteristic	Preserved Function-Stage 1 CKD	Stage 2 CKD	Stage 3 CKD	Stage 4-5 CKD	Dialysis	P Value*
(Mean±S.D.)	(N = 30)	(N = 5)	(N = 92)	(N = 27)	(N = 9)	
**Labs**						
Total cholesterol, mg/dL	160.6 ±32.9	160.4±42.9	166.6±41.6	147.2±40.6	147.4±42.9	0.42
Hemoglobin, g/dL	13.9±1.4	13.2±2.0	12.9±1.8	12.0±1.5	11.8±1.8	<0.001
Creatinine, mg/dL	0.8±0.1	1.0±0.2	1.6±0.4	3.3±2.0	7.1±3.4	<0.001
Blood urea nitrogen, mg/dL	14.5±3.8	20.2±5.8	30.3±10.7	55.6±28.5	42.1±12.9	<0.001
Estimated GFR, mL/min/1.73 m^2^	105.9±12.0	73.8±8.5	44.8±8.5	22.2±6.5	8.9±2.9	<0.001
Albumin, gm/dL	4.1±0.4	4.2±0.3	4.3±0.4	4.1±0.4	3.6±0.5	<0.001
Albumin:creatinine ratio, mg/G,	0.0 (152.3)	20.7 (433.0)	53.8 (216)	90.4 (184.1)		0.37
median (IQR)^1^						
Log albumin:creatinine, mg/G^1^	5.0	4.0±2.3	4.1±2.0	5.1±1.5		0.51

S.D. = Standard deviation., I.Q.R. = Interquartile Range,

*ANOVA for normally distributed continuous variables, chi-square test for -parametric trend test for non-normally distributed continuous variables, and chi-squared tests for count variables. ^1^N = 66: Preserved renal function n = 1, Stage 2 CKD n = 10, Stage 3 CKD n = 44, Stage 4/5 CKD n = 11.

Urinary albumin excretion was available for 66 patients. Logarithmically transformed albumin to creatinine ratio was 5.0± mg/G among those with preserved renal function, 4.0±2.3 in those with stage 2 CKD, and 4.1±2.0 mg/G in those with stage 3 CKD, and 5.1±1.5 in those with stage 4/5 CKD (P = 0.51).

### Endoglin Concentration and Renal Function

**Figure 1:**
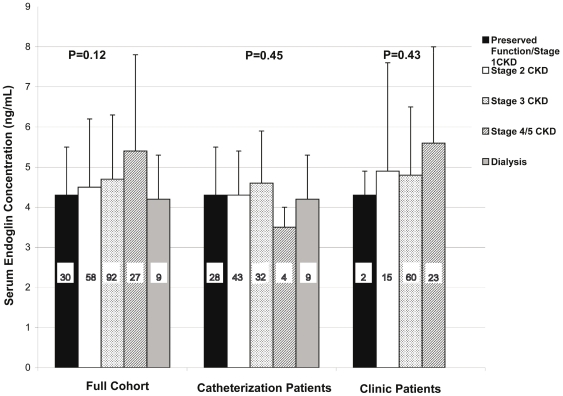
Endoglin concentration according to CKD class. Number of patients for each category is listed within the corresponding bar.

**Figure 2:**
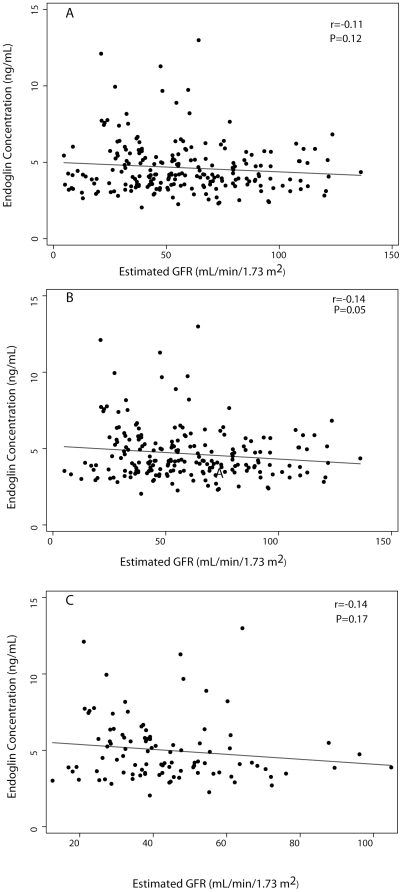
Scatter plots of endoglin concentration vs. estimated glomerular filtration rate. a) All comers b) Dialysis patients excluded c) Patients recruited in clinic.

Endoglin concentration did not vary significantly across categories of baseline renal function (**Figure 1**). Mean Endoglin concentration was 4.3±1.2 ng/mL among those with preserved renal function, 4.5±1.7 ng/mL in stage 2 CKD, 4.7±1.6 ng/mL in stage 3 CKD, 5.4±2.4 ng/mL in stage 4/5 CKD, and 4.2±1.1 ng/mL in individuals with ESRD, (P = 0.12). There was a non-significant (P = 0.09) trend consistent with an increase in endoglin across categories of CKD among the subgroup of subjects not on chronic dialysis. However, as shown in **Figure 2**, the correlation was weak (r = 0.12) and non-significant (P = 0.05). Within the preserved renal function/stage 1 CKD group, endoglin concentrations were not different in individuals with eGFR ≥110 mL/min/1.73 m^2^ compared with eGFR 90-<110 mL/min (4.65±1.32 ng/mL vs. 4.09±1.09 ng/mL, P = 0.23). Endoglin concentration was also similar among individuals recruited as transplant donors/stone patients compared to subjects with eGFR≥90 recruited for other reasons (4.24±0.74 ng/mL vs. 4.27±1.21, P = 0.95). Endoglin concentration also did not vary across categories of renal function when eGFR>110 mL/min/1.73 m^2^ (P = 0.16) or transplant donor/stone patient (P = 0.19) were considered as distinct categories of baseline renal function.

Although endoglin concentrations were marginally higher among individuals recruited from clinic (β = 0.67, P = 0.003, **Table 3**) than in individuals recruited from the catheterization lab, the association between endoglin concentration and class of renal function was qualitatively similar in both groups, and it was not significantly associated with CKD class, P = 0.45 and P = 0.43, respectively (**Table 4** **)**. The association between endoglin and CKD was also non-significant when the analysis was restricted to women (P = 0.21) or to the subgroup of individuals with diabetes (P = 0.28). Among other baseline and demographic characteristics, only the presence of diabetes (β = 0.47, P = 0.04) was significantly associated with circulating endoglin concentration. Neither total cholesterol nor baseline hemoglobin concentration was associated with circulating endoglin concentration. Although endoglin concentrations were lower in individuals with serum albumin concentrations less than the median value of ≤4.3 gm/dL compared with individuals with albumin > 4.3 gm/dL (4.4±1.5 ng/mL vs. 4.9±2.0 ng/mL), the difference was not significant, P = 0.07.

**Table 3:** Univariate Associations of Clinical Characteristics with Endoglin Concentration.

Characteristic	Unadjusted Β (95% CI)	P Value
**Demographics**		
Age		
≤40 years	Reference	Reference
40-60 years	-0.15	0.76
>60 years	-0.47	0.32
Male, n (%)	0.27	0.25
Black Race (vs. other race)	0.48	0.15
Hispanic	-0.41	0.21
Recruitment site, (clinic vs.	0.67	0.003
cardiac catheterization lab)		
**Physical Exam**		
Weight, kilogram, >88.6 vs. ≤88.6 Kg	0.31	0.31
Systolic blood pressure, per 10 mm Hg	0.01	0.37
Diastolic blood pressure, per quintile	0.12	0.15
**Past Medical History**		
Coronary disease	-0.11	0.71
Hypertension	0.13	0.64
Diabetes	0.47	0.04
Congestive heart failure	0.16	0.58
Obstructive lung disease	-0.15	0.78
Peripheral vascular disease	0.25	0.50
Hyperlipidemia	-0.12	0.64
Smoking	0.10	0.80
Cause of CKD		
Diabetes	1.35	0.001
Hypertension	1.27	0.002
Other/Unknown	Reference	Reference
**Labs**		
Total cholesterol, > 158 vs. ≤158 mg/dL	-0.27	0.37
Hemoglobin, >13 vs. ≤13 g/dL	0.29	0.23
Albumin, >4.3 vs. ≤4.3 g/dL	0.50	0.07

mg = milligram. G = gram. dL = deciliter. Kg = kilogram. CKD = Chronic Kidney Disease.

**Table 4:** Univariate Associations of Renal Function with Endoglin Concentration.

Characteristic	Unadjusted Β	P Value
	(95% CI)	
**Renal Function**		
Estimated GFR (per 10/mL/min/1.73m^2^)	-0.06	0.12
CKD Class (per 1 category change)^*^	0.16	0.09
CKD Category	--	--
Preserved Renal function/Stage 1 CKD	Reference	Reference
Stage 2 CKD	0.18 (-0.56, 0.92)	0.63
Stage 3 CKD	0.46 (-0.23, 1.15)	0.19
Stage 4-5 CKD	1.03 (0.15, 1.90)	0.02
Dialysis	-0.10	0.89

GFR = glomerular filtration rate CKD Classes include normal/stage 1 CKD, stage 2 CKD, stage 3 CKD, stage 4/5 CKD, and dialysis.

To understand whether the apparent lack of an association between renal function and endoglin concentration was confounded by other characteristics, we constructed a series of multivariable regression models as shown in **Tables 5** and **6**. Adjusting for age, sex, race, diabetes, hypertension, smoking, cholesterol, congestive heart failure, peripheral vascular disease, obstructive lung disease, and recruitment site attenuated the association between baseline renal function and circulating endoglin concentration. No significant associations between renal function and endoglin concentration were observed regardless of whether renal function was analyzed as a continuous (eGFR) or categorical (CKD class) predictor. Results were qualitatively similar after additional adjustment for Hispanic ethnicity and serum albumin concentration, and the association between renal function and endoglin concentration remained non-significant (P≥0.42) regardless of whether endoglin concentration was analyzed as a continuous or categorical outcome variable. There was no evidence for a significant interaction between renal function and diabetes or renal function and recruitment site in any of the models (Linear Model P_interaction_ = 0.53 and 0.17 for interactions between eGFR and diabetes and GFR and recruitment site, respectively).

**Table 5:** Adjusted Association of Glomerular Filtration Rate with Endoglin Concentration.

Model	Measure of Association (95% CI)	Significance
	per 10 mL/min/1.73 m^2^	
**Linear Regression**	**β**	**P Value**
Model 1	0.01 (-0.09, 0.11)	0.81
Model 2	-0.01 (-0.10, 0.09)	.91
**Categorical Regressions**	**Odds Ratio**	**P Value**
Ordered logistic regression	1.01 (0.91, .13)	0.85
Logistic regression	0.99 (0.87, 1.13)	0.90

Categorical models and linear Model 1 adjusted for age, sex, race, diabetes, hypertension, smoking, cholesterol, congestive heart failure, peripheral vascular disease, obstructive lung disease and recruitment site. Model 2 additionally adjusted for Hispanic ethnicity and serum albumin. CI = confidence intervals. CKD = chronic kidney disease.

**Table 6:** Adjusted Association of CKD Stage with Endoglin Concentration.

Model	Measure of Association (95% CI)	Significance
	per 10 mL/min/1.73 m^2^	
**Linear Regression**	**β**	**P Value**
**Model 1**		
Preserved Renal function/Stage 1 CKD	Reference	Reference
Stage 2 CKD	-0.04 (-0.82, 0.74)	0.93
Stage 3 CKD	-0.04 (-0.89, 0.81)	0.93
Stage 4-5 CKD	0.34 (-0.73, 1.40)	0.54
Dialysis	-0.08 (-1.48, 1.31)	0.91
**Model 2**		
Preserved Renal function/Stage 1 CKD	Reference	Reference
Stage 2 CKD	-0.00 (-0.77, 0.76)	0.99
Stage 3 CKD	-0.07 (-0.91, 0.76)	0.86
Stage 4-5 CKD	0.43 (-0.63, 1.49)	0.42
Dialysis	0.39 (-0.98, 1.77)	0.57
**Categorical Regressions**	**Odds Ratio**	**P Value**
**Ordered logistic regression**		
Preserved Renal function/Stage 1 CKD	Reference	Reference
Stage 2 CKD	1.05 (0.45, 2.45)	0.91
Stage 3 CKD	1.20 (0.48, 3.00)	0.70
Stage 4–5 CKD	1.28 (0.39, 4.24)	0.68
Dialysis	1.09 (0.24, 4.99)	0.92
**Logistic regression**		
Preserved Renal function/Stage 1 CKD	Reference	Reference
Stage 2 CKD	0.74 (0.28, 2.00)	0.54
Stage 3 CKD	0.96 (0.33, 2.78)	0.93
Stage 4–5 CKD	0.91 (0.23, 3.55)	0.89
Dialysis	1.78 (0.30, 10.58)	0.53

Categorical models and linear Model 1 adjusted for age, sex, race, diabetes, hypertension, smoking, cholesterol, congestive heart failure, peripheral vascular disease, obstructive lung disease and recruitment site. Model 2 additionally adjusted for Hispanic ethnicity and serum albumin. CI = confidence intervals. CKD = chronic kidney disease.

Results were qualitatively similar when eGFR was analyzed as a restricted cubic spline, and were not suggestive of an association between renal function and endoglin concentration (**Figure S1**).

### Endoglin Concentration and Albuminuria

**Figure 3:**
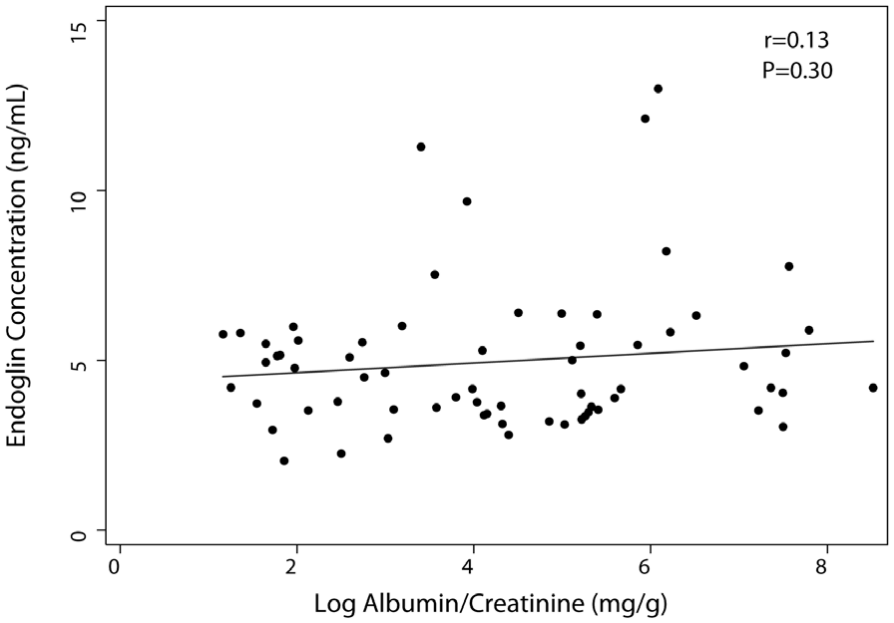
Association of endoglin concentration and urinary albumin excretion. Scatter plot of endoglin concentration vs. microalbumin to creatinine ratio.

Urinary albumin concentrations were higher among individuals with higher circulating endoglin concentrations (**Figure 3**). However, this correlation was weak and did not achieve statistical significance (r = 0.13, P = 0.30).

## Discussion

Changes in the regulation of angiogenesis appears to be a key factor in the pathogenesis of CKD and cardiovascular disease [1], [2], [3], [4], [5], [6], [7], [27], [28]. This suggests that increased concentrations of angiogenesis inhibitors may partly explain the markedly increased risk of developing and dying from cardiovascular disease in individuals with CKD. Endoglin, an ancillary TGF-β receptor, is a key player in angiogenesis, nitric oxide coupling and heart development that plays an important regulatory role in both renal and cardiovascular disease [20], [29], [30], [31]. We therefore hypothesized that an increase in soluble endoglin concentration as GFR declines might contribute to the progression of CKD or play a mechanistic role in the association of CKD with increased risks of developing and dying from cardiovascular disease.

In this study, we analyzed 216 individuals with various degrees of renal dysfunction and were unable to confirm an association between CKD and increases in the concentration of soluble endoglin. We found, in fact, that patients with ESRD, the most severe form of renal dysfunction, had on average the lowest circulating concentration of soluble of endoglin. Renal protein excretion, another indicator of renal disease, was also not associated with circulating endoglin concentration in the subset of patients in whom albuminuria was measured. Our data thus suggest that an elevation in the baseline concentration of soluble endoglin with progressive CKD is unlikely to play a causative role in the progression of CKD or the association of CKD with cardiovascular disease. They further suggest that, despite its well described role in the pathogenesis of preeclampsia [20], elevation in the baseline concentration of soluble endoglin is unlikely to underlie the increased risk of preeclampsia associated with CKD.

Experimental studies have consistently demonstrated that both glomerular and renal interstitial endoglin expression increase dramatically following induction of either acute or chronic renal dysfunction, regardless of the mechanism of renal injury [32], [33], [34], [35]. Similarly, a single human study demonstrated increased tissue expression of endoglin in the renal interstitium of diseased compared with healthy human kidneys [36]. The absence of an increase in soluble endoglin in our study suggests that the increases in tissue concentrations of endoglin following renal injury are not accompanied by concomitant release of cellular endoglin into the circulation.

To the best of our knowledge, ours is the first study to analyze the association of circulating endoglin concentration with renal function. The apparent lack of an association between either GFR or proteinuria and circulating endoglin concentration is surprising. Although our study was not designed to explore the mechanisms controlling the synthesis of endoglin, a low rate of release of membrane bound endoglin into the circulation as well as a differential regulation of the production of the soluble and membrane forms of endoglin may explain these contrasting findings.

Our findings are also notable in light of recent studies demonstrating an inverse correlation between eGFR and sFLT-1 and a direct correlation between sFLT-1 and proteinuria [18], [37]. These findings suggested the possibility that a universal, synchronous increase in the concentration of angiogenesis inhibitors might occur in CKD and predispose individuals with CKD to cardiovascular disease and preeclampsia. The present study, however, suggests a more complex biology with disparate associations between renal function and the various pro and anti-angiogenic factors.

Endoglin plays an important role in embryonic heart development [31], [38] and is also involved in post-embryonic maintenance of normal cardiovascular function. Endoglin expression on coronary artery endothelial cells appears to modulate the effects of angiotensin 2 on vascular remodeling [39], and it helps regulate vascular reactivity via improved coupling of eNOS activity [30], [40]. The expression of the soluble form is negatively regulated by heme-oxygenase-1 [41], which itself plays a key role in cellular responses to oxidative stress and ischemia-reperfusion injury [42]. Finally, in more general terms, studies of both plaque angiogenesis [14] and myocardial angiogenesis [12], [13] have strongly implicated angiogenesis-related factors (such as endoglin) as important players in cardiovascular pathophysiology.

On the basis of this evidence, important effects on cardiovascular homeostasis would be expected if renal injury induced changes in circulating endoglin concentrations comparable to the changes observed in intra-renal tissue levels. To our knowledge, however, the hypothesis that changes in soluble endoglin concentration partly underlie the high incidence of cardiovascular morbidity and mortality in individuals with CKD has not been previously investigated. The absence of significant increases in soluble endoglin levels in the setting of CKD in the current study, suggests that (despite the theoretical attractiveness of our initial hypothesis) that soluble endoglin is not an important factor in the etiology in the enhanced atherosclerosis and cardiovascular disease that accompanies CKD.

It should be noted, that when individuals with the most severe forms of CKD—those with ESRD—were excluded, our data was more suggestive of a trend towards increasing endoglin concentration with declining GFR. However, even within this subgroup, the differences across categories of CKD were small and did not achieve statistical significance. Nevertheless, additional studies including larger numbers of individuals with both ESRD and pre-dialysis CKD are needed to clarify the behavior of endoglin in the most advanced stages of CKD.

Other limitations of our study should be recognized. We were unable to measure GFR directly and thus estimated GFR on the basis of serum creatinine level at the time of recruitment. GFR estimating equations are susceptible to inaccuracy, and we cannot rule out the possibility that CKD status was misclassified in some cases. Such misclassification is unlikely to explain our findings since we excluded patients with overt acute kidney injury, recruited many patients from a renal clinic (in which individuals generally have overt/well-established CKD), and because endoglin concentrations were lowest in the group with ESRD in whom estimation of GFR was not needed to classify the CKD status. Another issue is that the numbers of individuals with ESRD and severely reduced GFR was small. Additional studies including larger number of patients with advanced renal disease are warranted to confirm our findings.

It should also be noted that our patients were recruited from 2 different settings within a single hospital. The single-center nature of our study may limit the generalizability of our findings to other populations with CKD, and our findings merit confirmation in populations from other centers. Soluble endoglin levels collected within a single, uniform setting within our center would likely have been more homogenous. However, our approach has the advantage of being generalizable to a fuller spectrum of CKD patients and presentations than would have been possible had we recruited patients at only a single site within the institution. Additionally, although absolute levels were somewhat different between the two subgroups, the relative change across categories of CKD was similar regardless of subgroup, and our findings were unchanged when recruitment site was included as a co-factor in multivariable analyses.

In conclusion, soluble endoglin has been implicated in the pathogenesis of renal and cardiovascular disease. To understand whether increases in the concentration of soluble endoglin underlie the strong associations of CKD with increased risks of developing cardiovascular death, we analyzed soluble endoglin concentrations in a large cohort of individuals with and without CKD and found no association between eGFR or urinary albumin excretion and soluble endoglin levels. Our findings suggest that increases in the baseline levels of soluble endoglin in individuals with CKD are unlikely to contribute to the progression of CKD or to explain CKD-associated cardiovascular disease.

## Supporting Information

Figure S1 Restricted cubic spline analysis of the association of GFR with endoglin concentration.
(TIF)
