# Supplementary figures and images for: Circulating Endoglin Concentration Is Not Elevated in Chronic Kidney Disease
Source: PLoS One. 2011 Aug 19;6(8):e23718. doi: 10.1371/journal.pone.0023718 (PMC3158786; doi:10.1371/journal.pone.0023718)

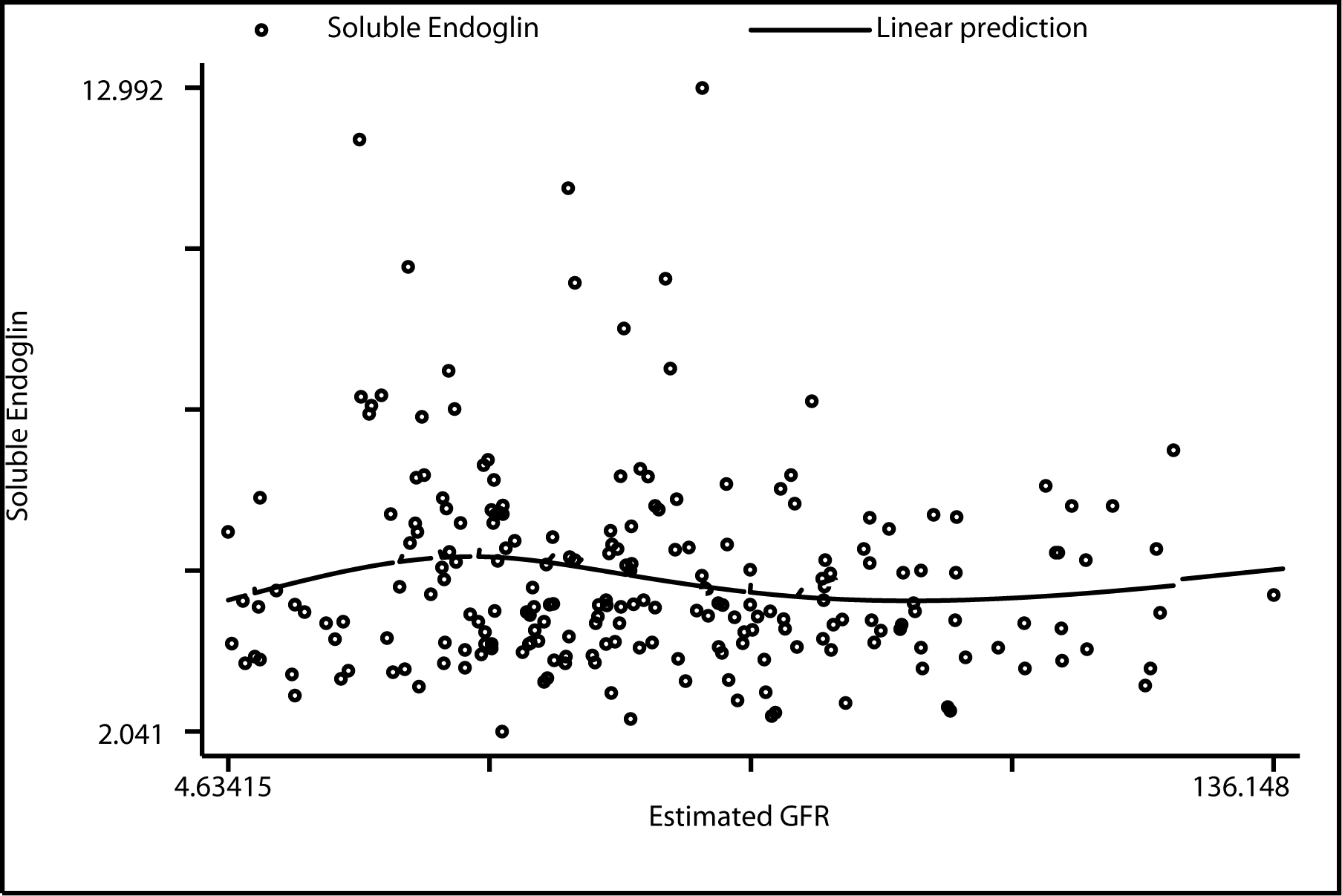

Supplement: Figure S1 — Restricted cubic spline analysis of the association of GFR with endoglin concentration. (TIF) [file pone.0023718.s001.tif]
